# Enhanced transanal surgery training through a 4K 3D surgical exoscope: a novel approach for transanal surgery

**DOI:** 10.1007/s00384-024-04739-z

**Published:** 2024-10-15

**Authors:** Antonino Spinelli, Leonidas Chardalias, Michele Carvello, Matteo Sacchi, Leandro Siragusa, Carlotta La Raja

**Affiliations:** 1 Department of Biomedical Sciences, Humanitas University, Via Rita Levi Montalcini 4, 20072 Pieve Emanuele, Milan Italy; 2 Division of Colon and Rectal Surgery, IRCCS Humanitas Research Hospital, Via Manzoni 56, 20089 Rozzano, Milan Italy; 3 2nd Department of Surgery, Aretaieion University Hospital, School of Medicine, National and Kapodistrian University of Athens, Athens, Greece

**Keywords:** Transanal surgery, Surgical exoscope, Surgical training, Pouch surgery, TTSS

## Abstract

**Purpose:**

Recently, exoscope was introduced as a more ergonomic alternative to microscope, mainly in nerve and spinal surgery. Exoscope use in general surgery is still experimental and just few reports are present in literature. Here, we describe for the first time its application in transanal surgery, specifically during the transanal transection and single-stapled anastomosis in ileal-pouch anal anastomosis.

**Methods:**

After completing the proctectomy and pouch formation laparoscopically, two surgeons performed the transanal transection and single-stapled anastomosis using the vision provided by the ORBEYE™ exoscope system with a 3D 4K orbital camera and a 55-inches 3D screen. The transanal procedure was carried out with the surgeons looking at the 3D screen rather than at the operating field.

**Results:**

The system subjectively provided excellent operative view thanks to the magnification capacity and the high resolution. The ergonomics was improved compared to classical transanal surgery, allowing the operators and observers to have the same view in a comfortable position. In particular, the exoscope magnified vision allowed for clearer demonstration of techniques to trainees.

**Conclusions:**

This is the first report on the intraoperative application of the ORBEYE™ surgical exoscope in transanal surgery. The magnified vision allowed precise movements and the system appeared potentially a ground-breaking tool for surgical training. The ability to project high-quality images to observers make it ideal for teaching complex transanal procedures**.** Further studies are encouraged to validate this approach into standard colorectal practice.

**Supplementary Information:**

The online version contains supplementary material available at 10.1007/s00384-024-04739-z.

## Introduction

The use of microscope for surgery traces back to the 1960s where it was primarily employed to help surgeons dealing with millimetric structures such as vessels and nerves. Microscope became well established in neuro and spine surgery, though some experiences were reported also in other surgical fields. Recently, exoscopes such as the ORBEYE™ (Olympus, Tokyo, Japan) were implemented to provide the same grade of magnification with the advantage of the 4K 3D vision and an improved ergonomic setting [1–3].

In general surgery, exoscope use has been described in few open abdominal operations, but never for transanal surgery where its application may help in magnifying a surgical field that is deep and narrow [4, 5]. The limitations in terms of visibility of traditional transanal surgery have also the relevant consequence that training in this field is often suboptimal. An exoscope 4K 3D imagery has the potential to enhance the ability of trainers to teach and demonstrate transanal techniques. We aimed to explore the potential training and ergonomics benefits of this novel technique, and here we present for the first time its application in transanal surgery, specifically during transanal transection and single-stapled anastomosis (TTSS) in ileal-pouch anal anastomosis (IPAA) [5].

## Methods

This study was performed in line with the principles of the Declaration of Helsinki. The IRCCS Humanitas Research Hospital Ethics Committee does not require ethical approval for a technical note. Patient informed consent for recording and sharing operative images was obtained. The operation was carried out at IRCCS Humanitas Research Hospital, Rozzano, Milan, Italy. The manuscript has been prepared in accordance with the IDEAL Reporting Guidelines for the Evaluation of Surgical Innovation [6]. The checklist for IDEAL Stage 1 is presented in Table S1.

### Operative procedure

**Fig. 1:**
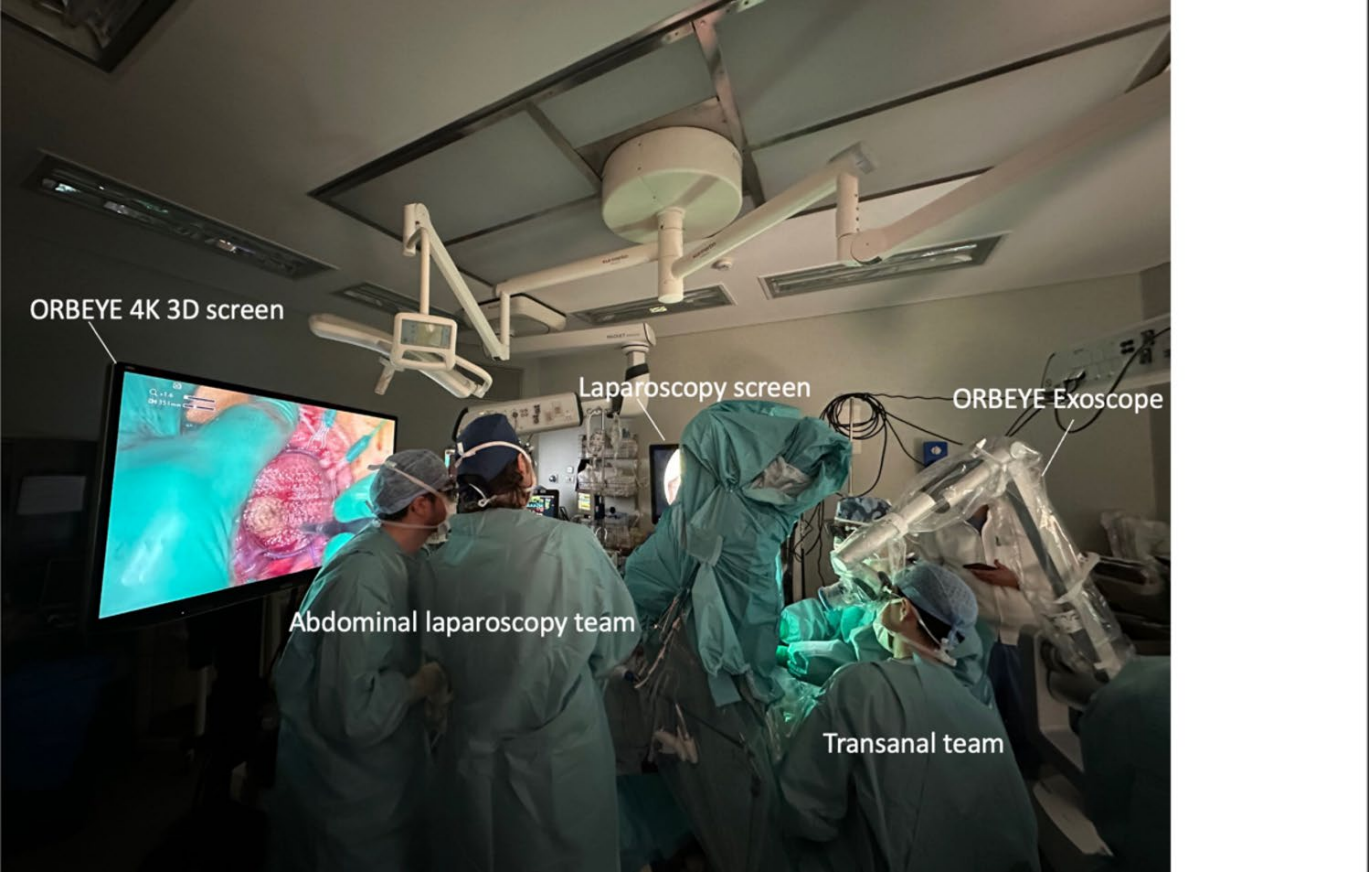
Theatre setup

We describe the use of the ORBEYE™ exoscope system (OLYMPUS, Tokyo, Japan) that includes a 3D 4K orbital camera and a 55-inch 3D screen. The camera is supported by a semi-robotic flexible arm, which connects an LED light source to an ultrafast image processor [7]. For transanal surgery using a surgical exoscope, the room setup is pictured in Fig. 1. The transanal procedure is carried out with the surgeon and assistant looking at the 3D screen rather than at the operating field, as it is usually done in laparoscopic surgery. The operators, as well as the observers present during the procedure, wear 3D glasses for 3D vision. During the transanal phase of the operation, all members of the team, including trainees not scrubbed, had an unobstructed view of the surgical field in all phases.

The technical aspects of the procedure are described in Video S1 that shows laparoscopic completion proctectomy and IPAA formation with TTSS and protective loop ileostomy performed in January 2024 in a 56-year-old male patient with ulcerative colitis (UC) referred to the Division of Colon and Rectal Surgery of Humanitas Research Hospital. One year before, the patient underwent urgent laparoscopic subtotal colectomy with end ileostomy for UC refractory to maximal medical therapy. He has a body mass index of 31, a past medical history of initial primary sclerosing cholangitis and atrial fibrillation, and an American Society of Anesthesiologists physical score of 2. One surgeon and two assistants completed the proctectomy laparoscopically and the pouch formation via the stoma incision. Subsequently, one experienced surgeon assisted by a junior surgeon completed the TTSS using the ORBEYE™ exoscope system for vision. This case was selected for demonstration because magnified vision in constructing a perfect IPAA with an adequate rectal cuff length can have technical and educational advantages. During the procedure the subjective ergonomic comfort of the two operators and the training potential of the system was evaluated.

## Results

The system subjectively provided excellent operative view thanks to the magnification capacity and the high resolution. The ergonomics was improved compared to classical transanal surgery, allowing both the operators and the observers to have the same view in a comfortable setting. The magnification of the surgical field facilitated a better understanding of the procedure for the observers. In particular, the clarity of visualization of the anal canal allowed an optimal demonstration on where to start placing the first purse string at the distal rectum, sparing the anal transition zone; and where to carry on the rectotomy in order to leave an adequate rectal cuff length.

The total operative time was 260 min. The patient was discharged on fourth postoperative day and underwent a pouchography as well as an endoscopic pouchoscopy 1 month after the operation which showed a regular anastomosis. The patient underwent a successful loop ileostomy closure 2 months after the primary operation.

## Discussion

This study reports on the first experience of exoscope application in transanal surgery during the TTSS IPAA for UC, specifically focusing on the implications for surgical training.

**Fig. 2:**
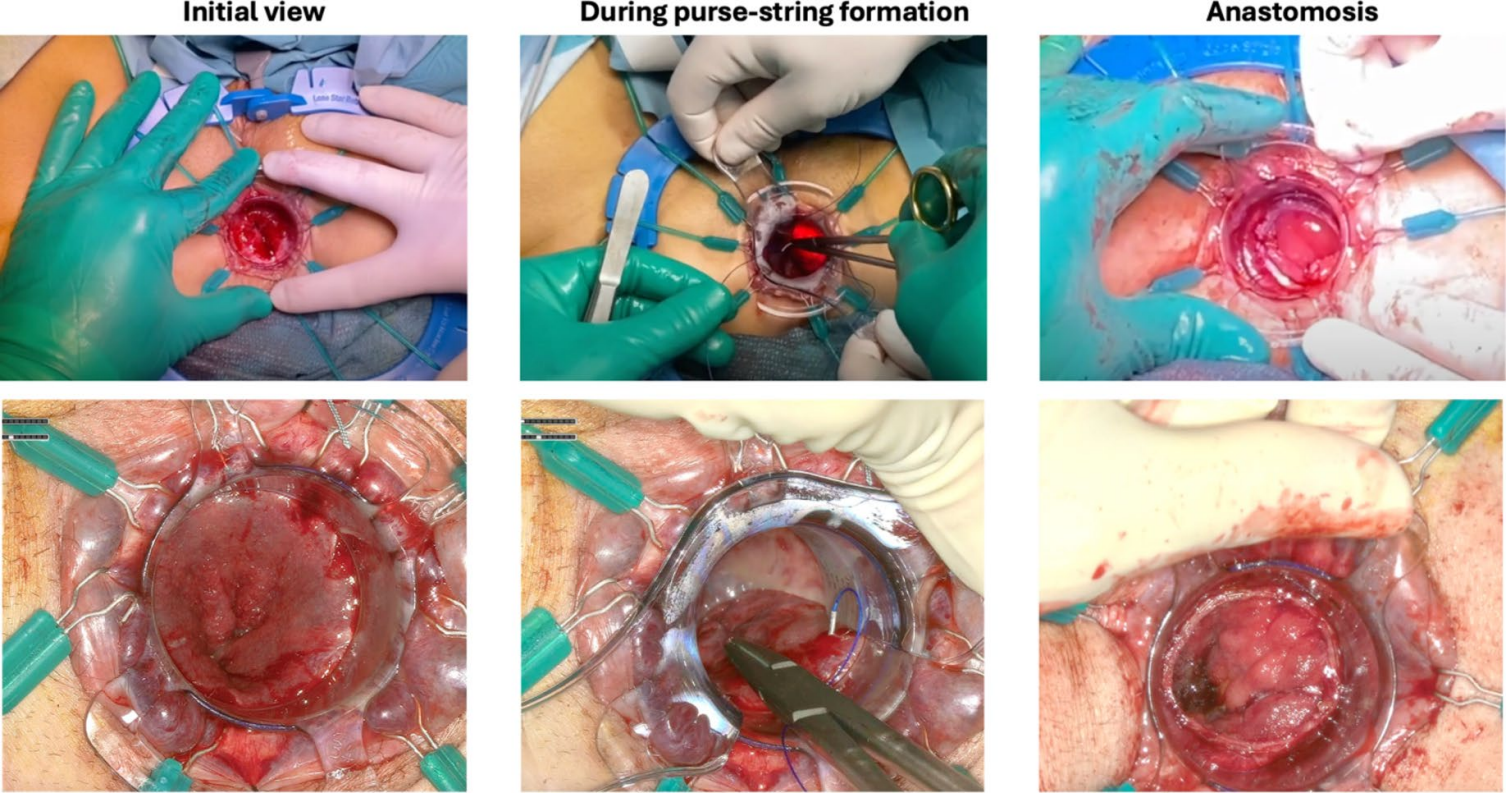
Comparison between standard operator’s view (top section) and a bidimensional reproduction of the 3D magnified exoscope vision (lower section) during ileal-pouch anastomosis formation with transanal transection and single stapled anastomosis

Both technical and educational advantages derive from the application of ORBEYE™ exoscope in transanal surgery. Having a good vision of the field in transanal surgery is intrinsically challenging: lighting can be suboptimal, and the assistant may have limited access to the field. With the use of ORBEYE™ exoscope, these limitations are overcome allowing all the operators and observers to have the same magnified vision with optimal and adjustable lighting (Fig. 2). Ergonomically more comfortable than traditional transanal surgery, ORBEYE™ camera angle can be adapted to the surgical field, while the surgeon and assistant look at the screen placed at the head of the patient in a natural seated position (Fig. 1).

The results relative to the comfort of ORBEYE™ use in general surgery was only evaluated by Corcione et al. in eight heterogeneous abdominal procedures, and it resulted generally positive [4], while the ergonomics in transanal surgery still needs to be evaluated systematically.

Operating transanally with the assistance of an exoscope differs from standard transanal surgery because the hand–eye coordination required is the one generally acquired in laparoscopy. The limited surgical field in transanal surgery has led over time to the development of different minimally invasive techniques to allow a laparoscopic-like approach to the distal rectum: from transanal endoscopic microsurgery (TEM) [8] to transanal minimally invasive surgery (TAMIS) [9] and then transanal total mesorectal excision (TaTME) [10]. Intuitively, this approach comes quite natural to minimally invasive surgeons, used to operate watching a screen rather than the operative field.

Until now, successful implementation of TTSS has been reached after attending dedicated in person training [11]. Beside operative advantages, this system could have relevance for surgical training. ORBEYE™ camera allows to deliver the same vision to a virtually unlimited number of people: not only real-time high-quality images are available for the surgical team and observers in the operating room, but the same images can be recorded and delivered—even in real time—to a remote audience. As exemplified in Video S1, the fixed, yet adjustable, image of the surgical field is extremely detailed and allows to observe the technique performed with great detail. Compared with traditional methods in transanal surgery, which may limit trainees’ understanding of the anatomy and surgical steps, the exoscope enhances learning opportunities. The high-definition visualization of the transanal surgical field may significantly shorten the learning curve, improving comprehension of the surgical anatomy. Additionally, this system has the potential to standardize high-quality training across different surgical teams or institutions through recorded or real-time sessions.

The cost of ORBEYE™ could be a limitation in its implementation. It must be taken into account, however, that there are not further operative costs beside the disposable arm cover and that the same device can be shared between different surgical services in the same hospital. A further limitation could be the need to rearrange the operatory room (OR) to fit the camera arm and a large screen, but this, in our experience, can be easily overcome as the ORBEYE™ camera arm is portable, not voluminous, and easily finds its setting also in a limited OR space.

No potential added risks compared to standard transanal surgery were identified during the use of the surgical exoscope.

## Conclusions

This is the first report on the intraoperative application of the ORBEYE™ surgical exoscope in transanal surgery. The system appeared potentially a ground-breaking tool not only for facilitating the access to a limited surgical field but also for revolutionizing surgical education. Enhanced visual clarity and ergonomic setup make the use of a surgical exoscope ideal for both teaching and performing transanal operations. Further studies are encouraged to validate this approach into standard clinical practice and cement its role in standardized training programs.

## Supplementary Information

Below is the link to the electronic supplementary material.

Supplementary Table S1 (PDF 148 KB)

Supplementary Video S1 (MP4 836965 KB)

## Data Availability

No datasets were generated or analysed during the current study.
