# Supplementary material for: Enhanced transanal surgery training through a 4K 3D surgical exoscope: a novel approach for transanal surgery
Source: Int J Colorectal Dis. 2024 Oct 15;39(1):163. doi: 10.1007/s00384-024-04739-z (PMC11480193; doi:10.1007/s00384-024-04739-z)
Supplement: Supplementary file 1 — Supplementary Table S1 (PDF 148 KB) [file 384_2024_4739_MOESM1_ESM.pdf]

IDEAL checklists for IDEAL Stages 1

Table S1

	Item	Checklist Item and example for key IDEAL items	
**STAGE 1**			
Title and Abstract	1a	**Identify the technique or device in the title, including IDEAL Stage 1 or 'first in human' in the title or abstract.**	Page 1 line 1-2
	1b	**Provide a structured summary of background, methods, results, and conclusions.**	Page 2 Lines 3-19
**Introduction**			
Background and objectives	2a	**Review of existing scientific literature, providing a clear explanation of the rationale for the new technique, including unmet clinical need.**	Page 3 Lines 3-12
	2b	**Details of pre-clinical development of the technique, including assessment of risks of failure and analysis of efforts to avoid harm.***	n/a
**Methods**			
Design	3	**Description of study design (e.g. case report or very small case series).**	Page 4 Line 18<br>Video S1
Participants	4a	**Transparent account of patient selection, with explicit detail about inclusion and exclusion criteria.**	Page 4 Lines 26-27
	4b	**Informed consent process described, including explanation of risks and acknowledgement of level of experience with technique/device. If informed consent is not obtained due to unplanned technique or modification, describe the discussion with the patient after the innovation. occurred.**	Page 4 Lines 4-5
	4c	**Setting, location, and timeframe of when and where the novel technique was performed, including hospital characteristics and appropriate details regarding the operator/team (e.g. experience).**	Page 4 Line 19<br>Page 4 Line 25

IDEAL checklists for IDEAL Stages 1

Intervention	5a	**Clear and detailed description of the new technique/device, including necessary pre- and post-procedure care.**	Page 4 Lines 10-17<br>Video S1
	5b	**Patient safety monitoring methods and safeguards.**	n/a
Outcomes	6	**Description of outcome measure(s) selected and how they were assessed, including patient reported outcome measures, if appropriate, utilising those measures that are standardised and validated, when available and applicable. When these are not available, provide rationale for the outcome measure(s) used.**	Page 4 Lines 27-29
**Results**			
Baseline Data	7	**Baseline demographic and clinical characteristics for each patient. Include how many patients were assessed for treatment and a description of which patients were included, excluded, or refused, and why (to be displayed in a flow diagram format, when appropriate).**	n/a
Intervention	8	**Technical feasibility of technique, including visual aids (e.g. photographs, videos, etc) when available.**	Page 6 Lines 3-8
Outcomes	9	**Appropriate clinical outcomes, including patient-reported outcome measures, when applicable.**	Page 6 Lines 9-11
Harms	10	**Transparent account of all harms or unintended effects reported for each patient.**	No unintended effect or patient harm to report
**Discussion**			
Stage End-Points	11	**Author's overall appraisal of the new technique, including discussion of risks and harms reported and suggestions to avoid them in future cases based on initial experience.**	Page 7 Lines 6-12 ; Lines 24-33 ; Lines 39-40
Conclusions	12	**Conclusions and relevance, including plans to progress to future IDEAL stages, or plans to discontinue further research.**	Page 9 Lines 3-7
**Other Information**			
Protocol	13	**Please quote reference or DOI if a protocol was written in advance and made available. If a protocol was not made available, consider including as a supplement if the journal allows**	n/a

Ethics	14	**Reference to ethical approvals obtained, and independent oversight, if applicable**	Page 2 Lines 28-29
Funding	15	**Sources of funding and support, role of funders, and other conflicts of interest**	Page 2 Lines 25-30
Regulatory Approvals	16	**Regulatory approvals being sought or obtained (e.g. CE Marking, FDA approval, etc) including the date of approval, if applicable**	n/a
